# Associations of serum vitamins levels with bone mineral density in the different race-ethnicities US adults

**DOI:** 10.1186/s12891-021-03997-0

**Published:** 2021-02-04

**Authors:** Xiang Li, Xun Liu

**Affiliations:** 1 Department of Orthopedics, Tianjin Fifth Central Hospital, Tianjin, 300450 China; 2 Department of Ultrasonics, Tianjin Fifth Central Hospital, No 41 Zhejiang Road, Tianjin, 300450 China

**Keywords:** Vitamin a, Serum folate, Vitamin B12, Vitamin C, Vitamin E, Bone mineral density

## Abstract

**Background:**

The conclusions on the associations of specific vitamin levels with bone mineral density (BMD) were controversial. Therefore, the aims of this study were to examine the associations of serum vitamins levels with BMD and the modified effect of race/ ethnicity on these associations in the US adults.

**Methods:**

This study was from the third National Health and Nutrition Examination Survey. All participants aged ≥18 years with complete data were eligible. Serum vitamins A, B9, B12, C, and E levels were assayed using the Quantaphase II Radioassay Kit (Bio-Rad). Dual-energy X-ray absorptiometry was employed to measure BMD, including femur neck and the total hip.

**Results:**

There were 6023 participants included in the final analysis. Serum folate, vitamins A and C levels were positively associated with BMD. No significant associations of serum vitamins B12 and E levels with BMD were observed. There were positive associations of serum folate level (*β* = 0.00027 and 0.00032; and *95% CI*: 0.00002–0.00057 and 0.00002–0.00063, respectively), vitamin A level (*β* = 0.01132 and 0.01115; and *95% CI*: 0.00478–0.01787 and 0.00430–0.01799, respectively), and vitamin C level (*β* = 0.00027 and 0.00029; and *95% CI*: 0.00012–0.00042 and 0.00013–0.00045, respectively) with BMD at femur neck and the total hip only in the Not Hispanic participants.

**Conclusion:**

Elevated serum folate, vitamins A and C levels were associated with a higher BMD. Furthermore, sex and race/ ethnicity modified the associations of serum vitamins levels with BMD.

## Background

As an important public health issue, osteoporosis is the most prevalent disease in women and the second one in men [1, 2]. The prevalence of osteoporosis was approximately 29.9% in females aged 50 and older in America, where half of nearly nine million fractures attributed to osteoporosis occur annually [3, 4]. In the USA, there were more than 2 million newly incident fractures annually, which caused the total costs of $16.9 billion [5]. By 2025, it was estimated that the total costs of osteoporotic fracture will be projected to $25.3 billion [6]. Therefore, improving BMD and reducing bone loss are essential and critical to prevent from osteoporosis.

It is well established that there is an important role of nutritional factors in bone mineral density (BMD) [7]. A number of literatures have declared that there were significant associations of dietary factors and nutrients with bone health [8, 9]. Previous studies found that deficiencies of Ca, Fe, vitamins A, K and D were positively associated with a higher risk of osteoporosis [8, 10]. Especially, vitamins have been identified as the main influence factors of BMD. However, the conclusions on the associations of specific vitamins with BMD were controversial [11, 12]. Furthermore, the existed studies focused on the associations of dietary intakes and supplements of vitamins with BMD. There were few studies to examine the associations of serum vitamins levels with BMD. On the other hand, a previous study declared that race-ethnicity was a major determinant of BMD [13]. Many studies also reported that hip fracture rates differed among varied races or ethnicities, such as African-American, Hispanic, and Caucasian humans in the USA [14–16]. However, little was known on the associations of serum vitamins levels with BMD across race/ethnic groups.

In view of the above mentioned facts, the aims of this study were twofold: to examine the associations of serum vitamins levels, including vitamins A, B9, B12, C, and E, with BMD in US adults; and to examine how sex and race-ethnicity modified the associations of serum vitamins levels with BMD. As a result, this study would provide additional evidence to improve BMD and prevent from osteoporosis, as well as assist individuals to benefit from faster screening for osteoporosis.

## Methods

All authors declared that all methods in this study were carried out in accordance with relevant guidelines and regulations.

### Study design

This study was conducted under the National Health and Nutrition Examination Survey. The purpose of this study was to examine the associations of serum vitamins levels with BMD at hip. Given both serum vitamins and BMD at hip were collected only in the third National Health and Nutrition Examination Survey (NHANES III), this study only used the data of the NHANES III. The NHANES III is designed to assess the health and nutritional status of residents in the US, and focused on oversampling many groups within the U.S. population aged ≥2 months. These oversampled groups included children aged 2 months to 5 years, persons over age 60, Mexican-American persons, and non-Hispanic black persons. The NHANES III interview includes demographic, socioeconomic, dietary, and health-related questions. The examination component consists of medical, dental, and physiological measurements, as well as laboratory tests administered by highly trained medical personnel. The detailed description of the NHANES III was published elsewhere [17]. This study was approved by the Institutional Review Board of the National Center for Health Statistics NHANES. All participants gave their written informed consent.

### Study population

**Fig. 1:**
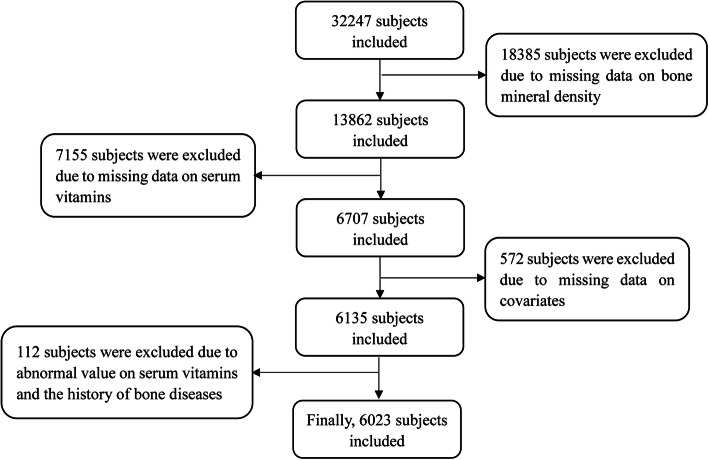
The flowchart of this study design

The included criteria were as follows: who were with complete data of interesting variables, such as BMD, serum vitamins, and other covariates, such as physical index, health behaviors, and medical histories; and who aged ≥18 years. The excluded criteria were as follows: who were with missing data in the interesting variables; or who had the history of bone diseases, such as arthritis and bone tumor. The flowchart is shown in Fig. 1.

All participants were interviewed to collect data, including demographic-socioeconomic index and health-related questions. Meanwhile, fasting blood samples were collected to assay the levels of serum vitamins.

### Measures

Serum samples from participants aged ≥18 years who were interviewed during phase II of this survey (1991–1994) in the NHANES III surplus sera project were used to assay serum vitamins concentrations including vitamins A, B9, B12, C, and E using the Quantaphase II Radioassay Kit (Bio-Rad) in the NHANES Laboratory of the Centers for Disease Control and Prevention. The coefficients of variation for vitamins A, B9, B12, C, and E in the NHANES III were 6, 7, 6, 4, and 5%, respectively. Furthermore, the intra-assay and inter-assay coefficients of variation were 5.2 and 2.5%, respectively. Details of the detection method are accessible at the NHANES website [18]. Dual-energy X-ray absorptiometry by using Hologic QDR 4500A fan-beam densitometers was employed to measure BMD at hip, including femur neck and the total hip BMD. The coefficients of variation were 3.2 and 2.4%, respectively. Details on the DXA examination protocol have been published elsewhere [13].

### Covariates

Height and weight were measured according to the standardized protocol, and used to calculate body mass index. All participants were divided into the White and Black as race, Mexican-American and Not Hispanic as ethnicity, or Non-Hispanic white, Non-Hispanic black, and Mexican-American as race-ethnicity. Urbanization classification based USDA Rural/Urban continuum codes was as follows: central counties or fringe counties of metro areas with ≥1 million population were considered as urban region; all other areas were considered as rural region. Health behaviors, such as smoking status, alcohol consumption, and physical activity, as well as medical histories, such as history of hypertension, history of diabetes, and history of fracture, were collected using a valid questionnaire.

### Statistical analysis

Since all continuous variables were normal distribution, means and standard deviations were used to express the distributions of continuous variables. Categorical variables were described by frequencies and percentages. Linear regressions were employed to examine the associations of serum vitamins levels with BMD at different sites and obtain the regression coefficients and 95% confidential intervals (*CIs*). Furthermore, linear regressions were stratified by race, ethnicity, age, and sex. In order to correct the confounding effects of covariates, age, sex, body mass index, smoking, alcohol consumption, physical activity, years of school completed, living regions, race, ethnicity, history of hypertension, history of diabetes, history of fracture, and sample design weights were adjusted. All analyses were conducted using SAS 9.4 (SAS Institute Inc., Cary, NC, USA.). A two-tailed *P* ≤ 0.05 indicated the statistical significance.

## Results

### The characteristics of all participants

There were 6023 participants included in the final analysis. The mean of age was 48.62 years. The averages of serum vitamins levels were 1.98 μmol/L for vitamin A, 15.67 nmol/L for folate, 358.75 pmol/L for vitamin B12, 41.25 mmol/L for vitamin C, and 26.60 μmol/L for vitamin E. And the averages of BMD were 0.83 g/cm^2^ and 0.95 g/cm^2^ at femur neck and the total hip, respectively. There were 2686 males, accounting for 44.60%. All characteristics are shown in Table 1.

**Table 1:** The characteristics of all participants (*N* = 6023)

Characteristics	Mean/Frequency	Standard deviation/Percentage (%)
Age (years)	48.62	18.92
Years of school completed (years)	11.12	3.85
Serum folate (nmol/L)	15.67	12.44
Serum vitamin B12 (pmol/L)	358.75	146.83
Serum vitamin C (mmol/L)	41.25	24.65
Serum vitamin A (μmol/L)	1.98	0.57
Serum vitamin E (μmol/L)	26.60	11.39
Body mass index (kg/m^2^)	27.42	5.66
Bone mineral density of femur neck (g/cm^2^)	0.83	0.17
Bone mineral density of total region (g/cm^2^)	0.95	0.18
Sex		
Male	2686	44.60
Female	3337	55.40
Race		
White	4164	69.13
Black	1859	30.87
Ethnicity		
Mexican-American	1484	24.64
Not Hispanic	4539	75.36
Race-ethnicity		
Non-Hispanic white	2687	44.61
Non-Hispanic black	1852	30.75
Mexican-American	1484	24.64
Smoking		
No	4479	74.36
Yes	1544	25.64
Alcohol consumption		
No	5230	86.83
Yes	793	13.17
Living regionss		
Urban	2825	46.90
Rural	3198	53.10
Physical activity		
No	3512	58.31
Yes	2511	41.69
History of fracture		
No	5489	91.13
Yes	534	8.87
History of hypertension		
No	2490	41.34
Yes	3533	58.66
History of diabetes		
No	1104	18.33
Yes	4919	81.67

### The associations of serum vitamins levels with BMD

Table 2 displays the associations of serum vitamins levels with BMD. Serum folate level was positively associated with BMD at femur neck and the total hip (*P* = 0.035 and 0.010; *β* = 0.00024 and 0.00023; and *95% CI*: 0.00002–0.00051 and 0.00005–0.00051, respectively). Similarly, serum vitamins A and C levels were significantly associated with BMD at femur neck and the total hip (vitamin A: *P* = 0.010 and 0.006; *β* = 0.00749 and 0.00837; and *95% CI*: 0.00176–0.01322 and 0.00240–0.01433, respectively) and (vitamin C: all *P* <  0.001; *β* = 0.00026 and 0.00025; and *95% CI*: 0.00012–0.00039 and 0.00011–0.00039, respectively). No significant associations of serum vitamins B12 and E levels with BMD were observed.

**Table 2:** The associations of serum vitamins levels with bone mineral density at different sites (*N* = 6023)^a^

Sites	Serum vitamins	*β*	*95% CI*	*P*
**Femur neck**				
	Serum folate	0.00024	0.00002–0.00051	**0.035**
	Serum vitamin A	0.00749	0.00176–0.01322	**0.010**
	Serum vitamin B12	0.00001	−0.00002-0.00002	0.971
	Serum vitamin C	0.00026	0.00012–0.00039	**< 0.001**
	Serum vitamin E	0.00013	−0.00016-0.00043	0.383
**Total region**				
	Serum folate	0.00023	0.00005–0.00051	**0.010**
	Serum vitamin A	0.00837	0.00240–0.01433	**0.006**
	Serum vitamin B12	0.00001	−0.00001 − 0.00003	0.461
	Serum vitamin C	0.00025	0.00011–0.00039	**< 0.001**
	Serum vitamin E	0.00028	-0.00003-0.00058	0.077

^a^In all sites, age, sex, body mass index, years of school completed, race, ethnicity, living regions, smoking, alcohol consumption, physical activity, history of hypertension, history of diabetes, history of fracture, and sample design weights were adjusted

### The associations of serum vitamins levels with BMD stratified by race and ethnicity

The results stratified by race are presented in Table 3. The associations of serum vitamins B12 and E levels with BMD were consistent with those of the total population. However, there were positive associations of serum folate level with BMD at femur neck and the total hip in the Black participants (*P* = 0.013 and 0.018; *β* = 0.00086 and 0.00082; and *95% CI*: 0.00018–0.00154 and 0.00014–0.00151, respectively) but not in the White participants. Significant associations of serum vitamin A level with BMD were observed at femur neck in the White participants (*P* = 0.012) and at the total region in the Black participants (*P* = 0.024). Serum vitamin C was associated with BMD at femur neck both in the White and Black populations (*P* = 0.011 and 0.017, respectively), and at total region only in the White population (*P* = 0.007).

**Table 3:** The associations of serum vitamins levels with bone mineral density at different sites stratified by race^a^

Sites	Serum vitamins	White (***N*** = 4164)			Black(***N*** = 1859)		
		*β*	*95% CI*	*P*	*β*	*95% CI*	*P*
**Femur neck**							
	Serum folate	0.00007	−0.00022-0.00035	0.636	0.00086	0.00018–0.00154	**0.013**
	Serum vitamin A	0.00590	0.00075–0.01258	**0.012**	0.01020	−0.00094-0.02134	0.073
	Serum vitamin B12	0.00001	− 0.00002-0.00003	0.715	−0.00001	− 0.00005-0.00003	0.612
	Serum vitamin C	0.00019	0.00004–0.00034	**0.011**	0.00036	0.00006–0.00066	**0.017**
	Serum vitamin E	0.00022	−0.00009-0.00053	0.158	−0.00049	−0.00126-0.00029	0.218
**Total region**							
	Serum folate	0.00006	−0.00024-0.00036	0.690	0.00082	0.00014–0.00151	**0.018**
	Serum vitamin A	0.00511	−0.00193-0.01215	0.155	0.01297	0.00171–0.02423	**0.024**
	Serum vitamin B12	0.00001	−0.00002 − 0.00004	0.474	0.00001	-0.00004-0.00004	0.954
	Serum vitamin C	0.00021	0.00006–0.00037	**0.007**	0.00029	−0.00001-0.00059	0.061
	Serum vitamin E	0.00030	−0.00003-0.00062	0.076	−0.00005	−0.00083-0.00073	0.903

^a^In all sites, age, sex, body mass index, years of school completed, ethnicity, living regions, smoking, alcohol consumption, physical activity, history of hypertension, history of diabetes, history of fracture, and sample design weights were adjusted

As stratified by ethnicity, both in the Mexican-American and Not Hispanic participants, there were no significant associations of serum vitamins B12 and E levels with BMD, which were comparable with the total results. There were positive associations of serum folate, vitamin A, and vitamin C levels with BMD at femur neck (*P* = 0.047, 0.001, and 0.001, respectively) and the total hip (*P* = 0.039, 0.001, and <  0.001, respectively) in the Not Hispanic participants but not in Mexican-American participants (Table 4).

**Table 4:** The associations of serum vitamins levels with bone mineral density at different sites stratified by ethnicity^a^

Sites	Serum vitamins	Mexican-American (***N*** = 1484)			Not Hispanic (***N*** = 4539)		
		*β*	*95% CI*	*P*	*β*	*95% CI*	*P*
**Femur neck**							
	Serum folate	0.00003	−0.00067-0.00073	0.925	0.00027	0.00002–0.00057	**0.047**
	Serum vitamin A	− 0.00740	− 0.01947-0.00467	0.229	0.01132	0.00478–0.01787	**0.001**
	Serum vitamin B12	0.00003	−0.00001-0.00007	0.157	−0.00001	−0.00003-0.00002	0.501
	Serum vitamin C	0.00015	−0.00013-0.00044	0.290	0.00027	0.00012–0.00042	**0.001**
	Serum vitamin E	0.00020	−0.00039-0.00080	0.500	0.00012	−0.00022-0.00045	0.504
**Total region**							
	Serum folate	−0.00032	−0.00104-0.00039	0.379	0.00032	0.00002–0.00063	**0.039**
	Serum vitamin A	−0.00134	− 0.01370-0.01101	0.831	0.01115	0.00430–0.01799	**0.001**
	Serum vitamin B12	0.00004	−0.00001-0.00008	0.086	−0.00001	−0.00003-0.00003	0.960
	Serum vitamin C	0.00001	−0.00025-0.00034	0.766	0.00029	0.00013–0.00045	**< 0.001**
	Serum vitamin E	0.00022	−0.00039-0.00083	0.474	0.00032	−0.00003-0.00067	0.077

^a^In all sites, age, sex, body mass index, years of school completed, race, living regions, smoking, alcohol consumption, physical activity, history of hypertension, history of diabetes, history of fracture, and sample design weights were adjusted

The results stratified by age are shown in Table 5. Significant associations of serum vitamin C level with BMD at femur neck and the total hip were observed both in the 20–49 years and 50–90 years groups (*P* = 0.003, 0.019, 0.013, and 0.025, respectively). There were positive associations of serum folate level with BMD at femur neck and the total hip only in the 50–90 years group (*P* = 0.022 and 0.041; *β* = 0.00018 and 0.00013; and *95% CI*: 0.00007–0.00028 and 0.00004–0.00023, respectively). Similarly, serum vitamin A level was significantly associated with BMD in the 50–90 years group at femur neck (*P* = 0.043; *β* = 0.00834; and *95% CI*: 0.00026–0.01642) and at the total region (*P* = 0.042; *β* = 0.00913; and *95% CI*: 0.00032–0.01793). In line with the total results, there were no significant associations of serum vitamins B12 and E levels with BMD both in the 20–49 years and 50–90 years groups.

**Table 5:** The associations of serum vitamins levels with bone mineral density at different sites stratified by age^a^

Sites	Serum vitamins	20–49 years (***N*** = 3364)			50–90 years (***N*** = 2600)		
		*β*	*95% CI*	*P*	*β*	*95% CI*	*P*
**Femur neck**							
	Serum folate	−0.00020	− 0.00072-0.00032	0.445	0.00018	0.00007–0.00028	**0.022**
	Serum vitamin A	0.00252	−0.00666-0.01170	0.590	0.00834	0.00026–0.01642	**0.043**
	Serum vitamin B12	−0.00001	−0.00004-0.00003	0.744	0.00002	−0.00001-0.00004	0.304
	Serum vitamin C	0.00032	0.00011–0.00054	**0.003**	0.00013	0.00006–0.00022	**0.019**
	Serum vitamin E	−0.00097	− 0.00154-0.00040	0.101	0.00009	−0.00028-0.00045	0.639
**Total region**							
	Serum folate	−0.00005	− 0.00055-0.00045	0.836	0.00013	0.00004–0.00023	**0.041**
	Serum vitamin A	0.00724	−0.00165-0.01612	0.111	0.00913	0.00032–0.01793	**0.042**
	Serum vitamin B12	−0.00001	− 0.00003-0.00003	0.923	0.00003	−0.00001-0.00007	0.062
	Serum vitamin C	0.00026	0.00005–0.00047	**0.013**	0.00023	0.00003–0.00044	**0.025**
	Serum vitamin E	−0.00023	−0.00079-0.00032	0.411	0.00019	−0.00021-0.00059	0.358

^a^In all sites, sex, body mass index, years of school completed, race, ethnicity, living regions, smoking, alcohol consumption, physical activity, history of hypertension, history of diabetes, history of fracture, and sample design weights were adjusted

Table 6 presents the associations of serum vitamins levels with BMD at femur neck and the total hip stratified by sex. In males, there were positively associations of serum folate, vitamin A and C levels with BMD at both femur neck (*P* = 0.014, 0.004, and 0.046; *β* = 0.00040, 0.01365, and 0.00022; and *95% CI*: 0.00010–0.00089, 0.00431–0.02298, and 0.00001–0.00044, respectively) and total region (*P* = 0.010, < 0.001, and 0.016; *β* = 0.00042, 0.01709, and 0.00028; and *95% CI*: 0.00018–0.00092, 0.00761–0.02657, and 0.00005–0.00050, respectively). However, in females, only serum vitamin C was associated with BMD at femur neck and total region (*P* = 0.001 and 0.002; *β* = 0.00029, and 0.00028; and *95% CI*: 0.00012–0.00045 and 0.00010–0.00045, respectively).

**Table 6:** The associations of serum vitamins levels with bone mineral density at different sites stratified by sex^a^

Sites	Serum vitamins	Males (***N*** = 2686)			Females (***N*** = 3337)		
		*β*	*95% CI*	*P*	*β*	*95% CI*	*P*
**Femur neck**							
	Serum folate	0.00040	0.00010–0.00089	**0.014**	0.00016	−0.00015- 0.00047	0.299
	Serum vitamin A	0.01365	0.00431–0.02298	**0.004**	0.00298	−0.00421-0.01018	0.417
	Serum vitamin B12	−0.00001	−0.00005-0.00002	0.432	0.00001	−0.00001-0.00004	0.304
	Serum vitamin C	0.00022	0.00001–0.00044	**0.046**	0.00029	0.00012–0.00045	**0.001**
	Serum vitamin E	−0.00007	−0.00054-0.00040	0.773	0.00030	−0.00008-0.00068	0.118
**Total region**							
	Serum folate	0.00042	0.00018–0.00092	**0.010**	0.00015	−0.00018-0.00048	0.365
	Serum vitamin A	0.01709	0.00761–0.02657	**< 0.001**	0.00316	−0.00442-0.01073	0.414
	Serum vitamin B12	0.00001	−0.00003-0.00004	0.728	0.00002	−0.00001-0.00005	0.191
	Serum vitamin C	0.00028	0.00005–0.00050	**0.016**	0.00028	0.00010–0.00045	**0.002**
	Serum vitamin E	0.00029	−0.00019-0.00077	0.232	0.00037	−0.00002-0.00077	0.065

^a^In all sites, age, body mass index, years of school completed, race, ethnicity, living regions, smoking, alcohol consumption, physical activity, history of hypertension, history of diabetes, history of fracture, and sample design weights were adjusted

## Discussion

This study aimed to investigate the associations of serum vitamins levels with BMD at hip in US adults. The results suggested that serum folate, vitamins A and C levels were positively associated with BMD at femur neck and the total hip. Furthermore, significant associations of serum vitamin C level with BMD were fully observed both in the White and Black participants. However, the associations of serum folate, vitamins A and C levels with BMD were fully observed only in the Not Hispanic, 50–90 years, and male participants.

The results implied that serum folate level was positively associated with BMD at femur neck and the total hip, which was consistent with previous studies [6, 17]. Like tetrahydrobiopterin, folate was a cofactor for the enzyme of nitric oxide synthase, which can promote the maintenance of bone density by helping to preserve optimal nitric oxide synthase activity in the bone cells [19]. Furthermore, choline played important role in skeletal muscle through fat and protein metabolism, inflammation, and autophagy [20]. Adequate serum folate level could properly modulate fat and protein metabolism, which in turn decrease fatty acid synthesis and promote muscle growth and function [20]. On the other hand, adequate dietary intake of folate could counteract inflammation, apoptosis, and autophagy via promoting intercellular homeostasis [20]. Thus, serum folate indirectly modulated bone metabolism via bone muscle cross-talk.

Several epidemiological studies reported that a higher of serum vitamin A level could improve bone health, which was in line with the findings of this study [21, 22]. The possible mechanisms might be summarized as follows: First, vitamin A could indirectly improve bone health by restraining the excessive secretion of parathyroid hormone, a higher level of which was suggested to result in poor bone health [23, 24]. Second, vitamin A can promote bone growth by means of affecting the growth hormone and stimulating the production of insulin-like growth factor 1, which are conducive to bone health [25]. Third, oxidative stress and reactive oxygen species can promote the development of osteoporosis. Whilst vitamin A has a property of against them. Therefore, in this respect, vitamin A was associated with a better BMD [26, 27].

**Fig. 2:**
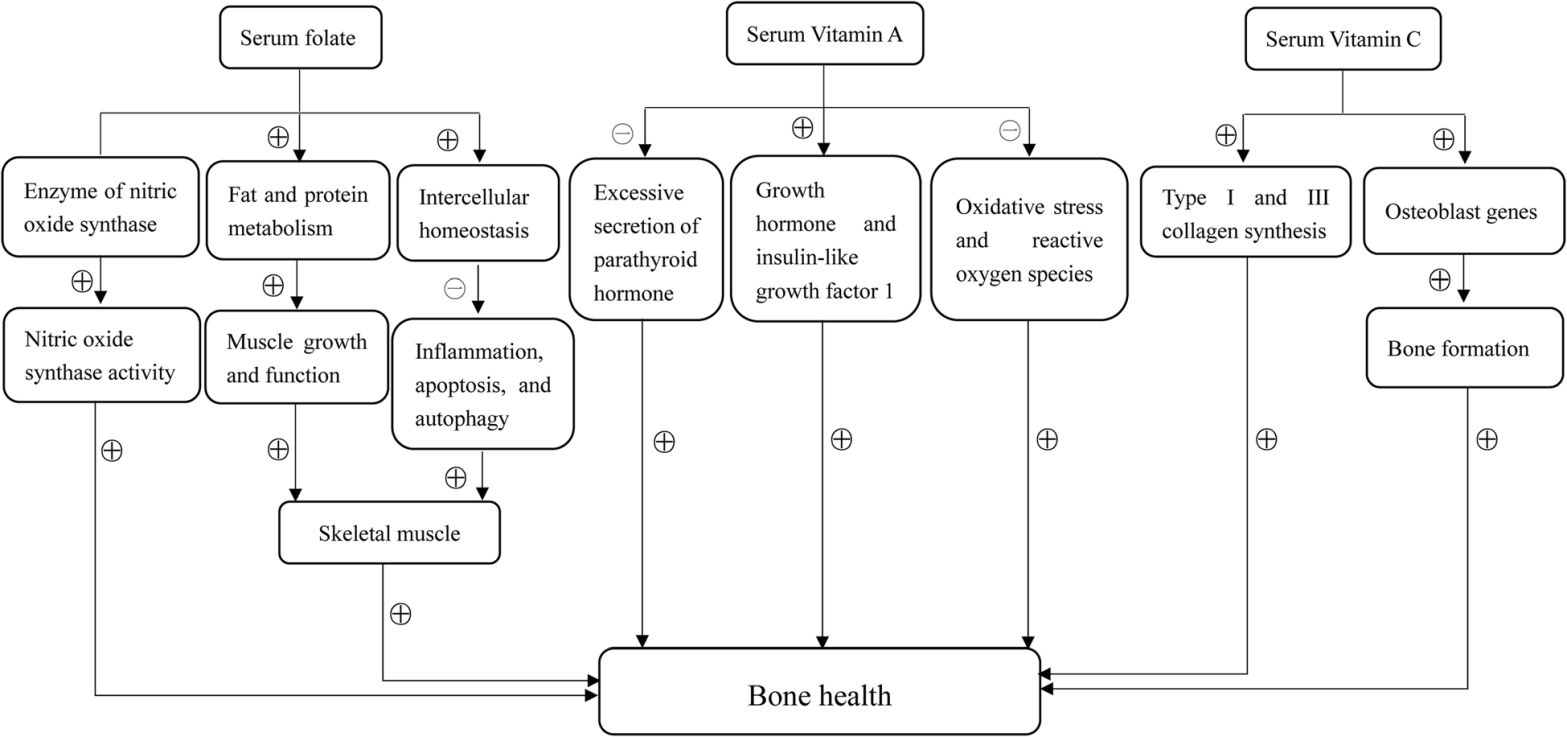
The possible mechanisms of serum folate, vitamins A and C on BMD

In this study, serum vitamin C level was associated with a better BMD, which was consistent with previous studies [28, 29]. It was confirmed that vitamin C involved in the collagen synthesis and can especially stimulate type I and III collagen synthesis [30, 31]. Furthermore, since vitamin C is essential for osteoblast genes, there was an active effect of vitamin C on bone formation by influencing expression of osteoblast genes and attenuating the loss of osteoblast differentiation markers [29]. Therefore, it was reasonable that vitamin C might improve bone health. In this study, there were no significant associations of serum vitamins B12 and E levels with BMD, which was consistent with previous studies [32, 33]. The potential mechanisms of serum folate, vitamins A and C levels on bone health are presented in Fig. 2.

Furthermore, it implied that race-ethnicity could modify the associations of serum vitamins levels with BMD in this study. Especially, serum folate level was associated with BMD at three site only in the Black participants. Significant associations of serum folate, vitamins A and C levels with BMD were observed only in the Not Hispanic participants. Many studies have reported that the Black subjects exhibited higher BMD than the White subjects at the femoral neck [14, 16, 34]. Meanwhile, a previous study declared that a lower hip strength was found in the Hispanic subjects compared with the White or Black subjects [35]. Furthermore, there were significant differences in the weight, body mass index, lean mass, and fat mass across races/ ethnicities, which could contribute to the differences of BMD [36]. Furthermore, the similar study from the NHANES III reported that the Mexican-America women had a higher femoral neck BMD and shorter hip axis length compared with the non-Hispanic White women in the USA [37]. Therefore, it was feasible that the race-ethnicity indeed affected the associations of serum vitamins levels with BMD.

In this study, it was found that there were positively associations of serum folate, vitamins A and C levels with BMD, especially in the 50–90 years and male population. The possible reasons might be that the nutrition status was worse in the older population than that in the young population. The prevalence of malnutrition and vitamins A and C deficiency significantly increased over age [38]. Therefore, the effects of serum vitamins levels on BMD might be more pronounced in the older population. Furthermore, serum vitamins levels and BMD in the elderly males were significantly higher than that in females, which might explain the sex difference in the associations of serum folate, vitamins A and C levels with BMD [38, 39].

### Strengths and limitations

There were some strengths in this study. First, this study comprehensively evaluated the associations of serum vitamins levels with BMD at hip in the US adults. Furthermore, some major covariates were adjusted in this study, such as race/ ethnicity, history of fracture, BMI and sample design weights. Therefore, this study would provide accurate evidence on the associations of serum vitamins levels with BMD. Second, differing from other studies to use dietary vitamins intake or supplement, serum vitamins levels were used in this study. Given the complicated relationship between dietary vitamins intake and serum level, serum vitamins levels seemed to better reflect the biological availability and activity of vitamins. Third, this study further examined the modified effect of race and ethnicity on the associations of serum vitamins levels with BMD. As a result, it would provide a better understanding on the important role of race-ethnicity in the associations of serum vitamins levels with BMD.

However, there were some limitations to be stated. First, this study was based on the NHANES III, in which the study population were restricted to the US subjects. Furthermore, the population of this study were relatively young. Therefore, it should be cautious when generalizing the results to older populations or populations outside the USA. Second, this study was a cross-sectional study, which was poor to examine the causal relationship between serum vitamins levels and BMD. A well designed cohort study should be conducted to confirm the findings of this study in the future. Third, since the complicated backgrounds of different races or ethnicities, the mechanisms of race/ ethnicity modifying the associations of serum vitamins levels with BMD failed to be fully explained in this study.

## Conclusions

Serum folate, vitamins A and C levels were positively associated with BMD at femur neck and the total hip. Furthermore, age, sex, and race/ ethnicity modified the associations of serum vitamins levels with BMD. Significant associations of serum vitamins levels with BMD were fully observed in the Not Hispanic, 50–90 years, and male participants. Therefore, this study would provide additional evidence to improve bone health, improve understanding the role of age, sex, and race/ethnicity in the associations of serum vitamins levels with BMD, and provide suggestions in public health interventions.

## Data Availability

The datasets analyzed during the current study are available in the website of the NHANES: https://www.cdc.gov/nchs/index.htm.

## Abbreviations

BMD: Bone mineral density
NHANES III: The third National Health and Nutrition Examination Survey:
*CIs*: Confidential intervals
